# A carnosine intervention study in overweight human volunteers: bioavailability and reactive carbonyl species sequestering effect

**DOI:** 10.1038/srep27224

**Published:** 2016-06-06

**Authors:** Luca Regazzoni, Barbora de Courten, Davide Garzon, Alessandra Altomare, Cristina Marinello, Michaela Jakubova, Silvia Vallova, Patrik Krumpolec, Marina Carini, Jozef Ukropec, Barbara Ukropcova, Giancarlo Aldini

**Affiliations:** 1 Department of Pharmaceutical Sciences, Università degli Studi di Milano, Milan, Italy; 2 Monash Centre for Health, Research and Implementation, School of Public health and Preventive Medicine, Melbourne, Australia; 3 Diabetes and Vascular Medicine Unit, Monash Health, Locked Bag 29, Clayton, VIC 3168, Australia; 4 Institute of Pathological Physiology, Faculty of Medicine, Comenius University, Bratislava, Slovakia; 5 Institute of Experimental Endocrinology, Biomedical Research Center, Slovak Academy of Sciences, Bratislava, Slovakia

## Abstract

Carnosine is a natural dipeptide able to react with reactive carbonyl species, which have been recently associated with the onset and progression of several human diseases. Herein, we report an intervention study in overweight individuals. Carnosine (2 g/day) was orally administered for twelve weeks in order to evaluate its bioavailability and metabolic fate. Two carnosine adducts were detected in the urine samples of all subjects. Such adducts are generated from a reaction with acrolein, which is one of the most toxic and reactive compounds among reactive carbonyl species. However, neither carnosine nor adducts have been detected in plasma. Urinary excretion of adducts and carnosine showed a positive correlation although a high variability of individual response to carnosine supplementation was observed. Interestingly, treated subjects showed a significant decrease in the percentage of excreted adducts in reduced form, accompanied by a significant increase of the urinary excretion of both carnosine and carnosine-acrolein adducts. Altogether, data suggest that acrolein is entrapped *in vivo* by carnosine although the response to its supplementation is possibly influenced by individual diversities in terms of carnosine dietary intake, metabolism and basal production of reactive carbonyl species.

Reactive Carbonyl Species (RCS) are electrophiles deriving from the oxidation of lipids and sugars. From a chemical point of view they are toxic aldehydes or ketones able to covalently modify proteins, nucleic acids and phospholipids forming irreversible adducts^1^,^2^.

RCS and their corresponding adducts with proteins have been studied by many research groups, in order to identify biomarkers of oxidative damage. As a matter of fact, RCS are nowadays considered also as potential targets for the development of a new class of drug^2^,^3^ since recent findings suggest a pathogenic role of toxic endogenous aldehydes in several chronic diseases including diabetes, atherosclerosis, some neurological disorders and cancer^1^,^4^,^5^.

In physiological conditions, RCS are efficiently detoxified through metabolic bio-transformations with aldehyde dehydrogenases (ALDHs) mainly involved in phase I metabolism, while glutathione conjugating enzymes have a pivotal role in phase II metabolism^6^,^7^. Recently, the importance of histidine containing peptides acting as phase II substrates has also been recognized. In fact, it has been demonstrated that carnosine (CAR) covalently binds RCS forming unreactive covalent adducts. Moreover, similar reactivity has been found for carnosine-related compounds (e.g. anserine) generally called histidine dipeptides. The efficacy of this class of compound was first evaluated *in vitro* by monitoring the disappearance of the UV signal generated by the RCS in presence of histidine dipeptides^8^. Then, more complex experiments based on LC-UV and ESI-MS allowed the characterization of the reaction mechanisms and products for most of the known RCS^9^,^10^.

Furthermore, carnosine activity have also been proven in oxidized rat muscle confirming that the RCS trapping activity also occurs in biological media and carnosine-RCS adducts can be considered as biomarkers of lipid peroxidation^11^. Finally, histidine dipeptides have been found to have a protective role against protein carbonylation induced by RCS in human and rat cell cultures^12^,^13^.

Concerning *in vivo* and *ex vivo* studies, histidine dipeptides have been tested in different animal models including the Zucker rat, which is a model of metabolic syndrome. Such experiments have allowed the identification, characterization and quantitation of carnosine adducts with some RCS by using mass spectrometry. Interestingly, it has been found that carnosine treatment correlates with an increase of the urinary excretion of adducts (e.g. CAR-HNE-MA, CAR-DHN and 3-HPMA), along with a decrease of urinary markers of protein carbonylation (e.g. AGE and ALE)^14^.

The sequestering activity of histidine dipeptides designed to be stable to carnosinases (i.e. enzymes responsible for carnosine degradation) have also been confirmed in other animal models^15^,^16^. However it is still not clear whether this protective effect is only due to a direct reaction with toxic aldehydes or it involves other pathways such as interaction with the receptor for advanced glycoxidation end-products (RAGE).

The first paper reporting the identification of carnosine-RCS adducts in humans was published by Baba and colleagues^17^. Urine samples from healthy, non-smoker adults were analyzed by LC-MS with the aim of identifying carnosine metabolites. The most abundant species identified were carnosine-propanol (m/z 285), carnosine-propanal (m/z 283), histidine-propanol (m/z 214) and histidine-propanal (m/z 212), which are the products of acrolein adduction on carnosine followed by metabolic transformations (i.e. reduction and hydrolysis). The vast majority of the metabolites (about 75%) have been identified as reduced acrolein adducts, suggesting that reduction is an important step of the metabolism of such adducts. However, the origin of the histidine conjugates is still not clear since they can arise either from the direct reaction between histidine and acrolein or from the hydrolysis of carnosine and protein adducts. Interestingly, HNE adducts have also been found but in lower amounts. Specifically, Baba and colleagues reported a urinary concentration of histidine-HNE (m/z 310) 75-fold lower than histidine-propanal and only trace levels of carnosine-HNE (m/z 383) and its reduced form CAR-DHN (m/z 385) have been detected. It is important to highlight that such adducts were detected in specimens collected from healthy volunteers, whereas no data have been reported so far for pathological conditions characterized by an expected increase of HNE levels.

Overall, these studies give convincing evidence that carnosine detoxifies RCS both in rodents and humans leading to interesting beneficial effects due to a decrease of protein carbonylation. Moreover, in humans, this activity has been detected despite the presence of hydrolytic enzymes (i.e. carnosinases) able to prevent prolonged carnosinemia.

Based on these evidences a carnosine intervention study in overweight/obese human subjects is herein reported. We chose to study overweight/obese individuals since obesity is known to be associated with an increase of oxidative stress, insulin resistance and the carbonylation of a number of adipose-regulatory proteins that may represent a mechanistic link between increased oxidative stress and the development of insulin resistance^18^.

In this study, we administered carnosine (2 g/day) orally for 12 weeks in order to evaluate the bioavailability and metabolic fate of carnosine in overweight and obese subjects and to demonstrate its ability to detoxify RCS by a direct trapping mechanism. To our knowledge, this is the first carnosine intervention study in overweight/obese human subjects.

## Results

### Preparation and purification of carnosine adducts *in vitro*

**Figure 1:**
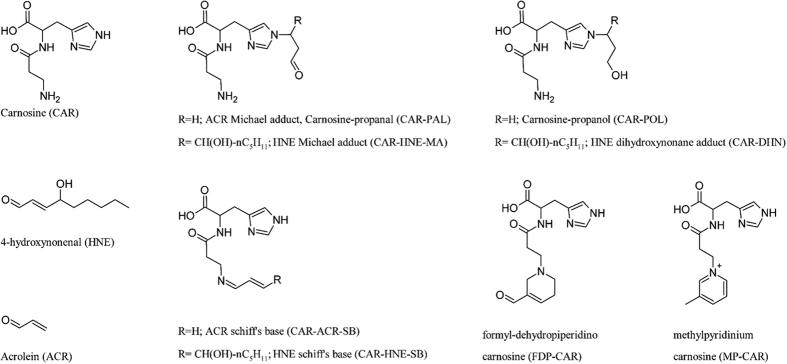
Structure of carnosine and its corresponding adducts with acrolein and 4-hydroxynonenal.

The concentrations of reaction products obtained from the *in vitro* reaction of carnosine with acrolein and HNE are reported in Table S1. The corresponding structures are reported in Fig. 1.

In detail, acrolein produced (3-formyl-3,4-dehydropiperidino) carnosine (FDP-CAR), carnosine-acrolein-aldimine (CAR-ACR-SB), carnosine acrolein Michael adduct (carnosine propanal) and (methylpyridinium) carnosine (MP-CAR).

On the other hand, only two adducts were formed when carnosine was incubated with HNE, namely carnosine-4-hydroxynonanal Michael adduct (CAR-HNE-MA) and carnosine-HNE-aldimine (CAR -HNE-SB).

As expected, the NaBH_4_ treatment induced the complete reduction of the double bonds for aldimine, Michael and 3-formyl-3,4-dehydropiperidino adducts.

The reaction of acrolein and HNE with carnosine induced a consumption of the initial amount of aldehyde equal to 95% and 93%, respectively.

### Preliminary screening of biological specimens for adduct identification

The biological specimens collected from the intervention study were analyzed by the method described in method section “Qualitative method for carnosine adducts identification by LC-ESI-MSMS” in order to identify the analytes of interest (i.e. carnosine and corresponding adducts). Neither carnosine nor any of the expected adducts were detected in plasma samples. On the other hand, carnosine, carnosine-acrolein Michael adduct (carnosine propanal) and its corresponding reduced form (carnosine-propanol) were detected in all urine samples, while neither the predicted HNE adducts nor other acrolein adducts like FDP-CAR were detectable.

**Figure 2:**
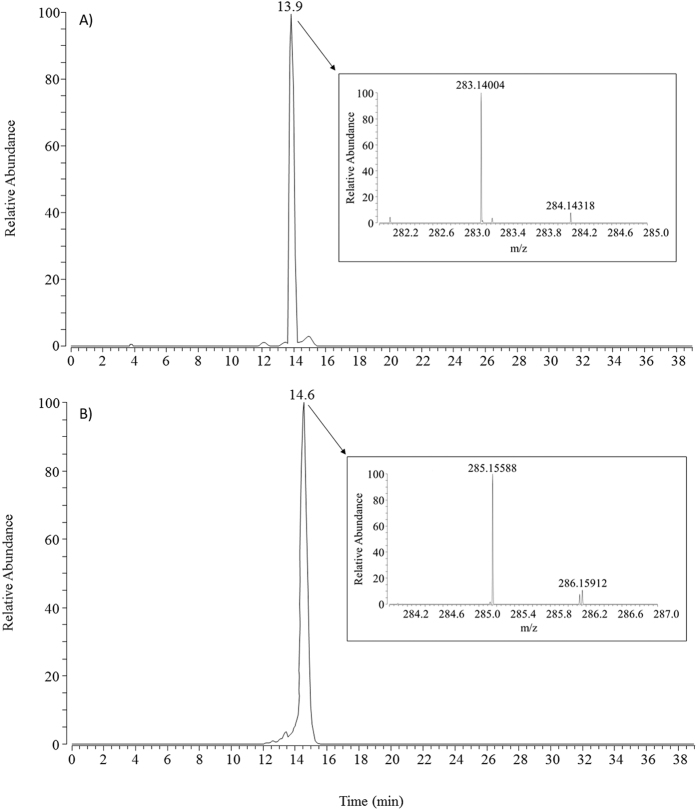
Single ion chromatograms (SICs) with corresponding mass spectra for carnosine-propanal adduct (chromatogram **A**) and carnosine-propanol adduct (chromatogram **B**) in the urine of subject #4.

The identification of such adducts was based on their retention time, accurate mass and fragmentation pattern. Figure 2 reports the extracted single ion chromatograms (SICs) for carnosine adducts detected in the urines from one subject involved in the study (subject #4). Extraction windows were 283.14008 ± 5 ppm for carnosine-propanal (Fig. 2A) and 285.15573 ± 5 ppm for carnosine-propanol (Fig. 2B).

Both chromatograms are characterized by a single peak eluting at retention times superimposable on those of the corresponding adducts prepared *in vitro* and as reported in Fig. 2 the experimental m/z values are shifted by 0.14 ppm and 0.53 ppm in respect to the theoretical values for carnosine-propanal and carnosine-propanol, respectively.

**Figure 3:**
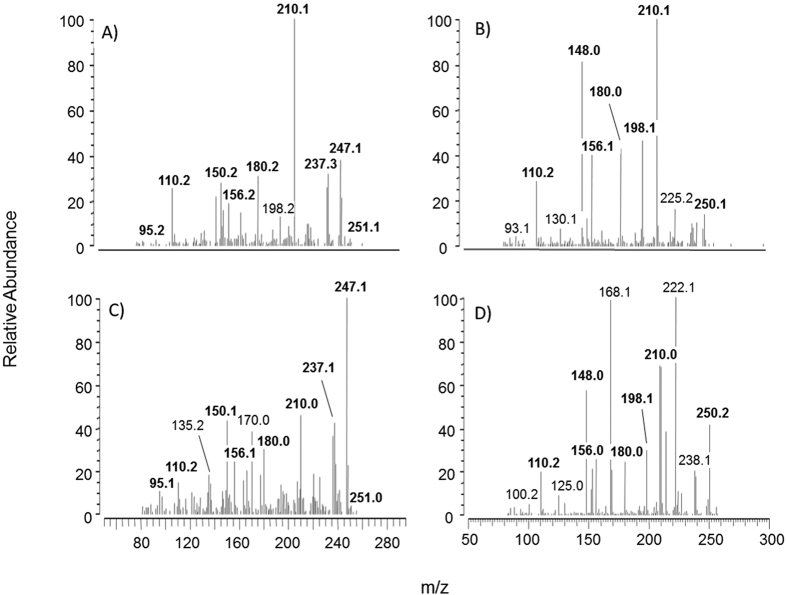
Tandem mass spectra (MSMS) for carnosine-propanal adduct detected *ex vivo* (spectrum **A**) or synthesized *in vitro* (spectrum **C**) and for carnosine-propanol adduct detected *ex vivo* (spectrum **B**) or synthesized *in vitro* (spectrum **D**).

Moreover, we also found similarities between the fragmentation patterns of synthesized adducts and the corresponding species detected *ex vivo*. As reported in Fig. 3, the spectra of adducts found *ex vivo* looks different compared to the corresponding spectra of synthesized adducts, especially concerning the relative abundance of fragments. Such differences could be due to a different isomeric composition of adducts synthesized compared to adducts detected *ex vivo*. However, we considered beyond the scope of the present paper to more deeply investigate the exact stereochemistry of the adducts since the present paper is focused on collect more clues about the fate of orally administered carnosine and its contribution, if any, to the mitigation of the carbonyl damage in overweight subjects.

### Carnosine and corresponding adducts calibration curves

Calibration curves were built both for carnosine and for adducts identified in the preliminary screening (i.e. carnosine-propanal and carnosine-propanol). The curves were prepared by spiking known amount of synthesized standards in aliquots in blank matrix (i.e. urine and plasma) collected from six healthy volunteers. A preliminary screening of the matrices used for preparing calibration curves revealed detectable amounts of carnosine and corresponding acrolein adducts in the urines, while no traces were detectable in plasma. In order to obtain urine with a lower basal level of analytes the healthy volunteers followed three days of ovo-lacto-vegetarian diet, which was able to considerably reduce the amount of carnosine and adducts. The specimens collected after the ovo-lacto-vegetarian diet were then used for preparing urine calibration curves. Concerning the internal standard, preliminary screening of blank urine and plasma confirms that it was not detectable.

The urinary LLOQs were 0.50 μM for carnosine, 0.10 μM for carnosine-propanal and 0.20 μM for carnosine-propanol; the LLOD were 0.20 μM for carnosine and 0.03 μM for both adducts. The amount of carnosine, carnosine propanal and carnosine propanol still detectable after the ovo-lacto-vegetarian diet was below the corresponding LLOQ and therefore it did not interfere with the calibration curve.

The method was found to be specific, precise and accurate according to the FDA guidelines: The CV % range from 5 to 15% and the bias didn’t exceed ± 15%.

The stability of adducts and internal standard was assessed as well by leaving the samples at 5 °C for 24 hours. The variance of the area ratio between the analytes and the internal standard was below 5%.

The intra and inter-day precision (CV%) and the accuracy of the method were determined on quality control samples containing carnosine and prepared separately from calibration standards, by analyzing five replicates at three concentration levels. Major details about the precision and accuracy are reported in Table S2 (supplementary material). Carnosine was found to be stable at 4 °C at least for 18 hours both in urine and plasma.

### Parameters determined in urine samples of overweight/obese individuals

The samples collected from all subjects before the intervention were used as control group (n = 29), while the samples collected after the intervention were grouped into two categories: subjects who received the placebo (n = 14) and subjects who received carnosine (n = 15).

The mass spectrometric method described in method section “Carnosine and adducts quantitation by LC-ESI-MS” allowed the quantitation of urinary levels of Carnosine (U-CAR), carnosine-propanal (U-PAL) and carnosine-propanol (U-POL). Urinary creatinine (U-CRE), total protein (U-PRO) and advanced glycoxidation end-products (U-AGE) were measured by spectrophotometric methods (see method section). The results are reported in Table S3 (supplementary information).

The correlations between variables were firstly studied using multivariate statistics. As reported in Table 1, Principal Component Analysis output gave a two-component solution explaining 72% of total data variance, with Principal Component 1 (PC1) alone explaining 50% of the total variance. PC1 shows positive loadings for all variables, suggesting that relations among different amounts of analytes are common across all samples.

This component describes the overall increase of analyte concentration correlated to an increase of creatinine excretion. This result is not surprising considering that creatinine is a marker of kidney glomerular filtration, which influences the urinary concentration of any excreted molecule.

Conversely, PC2 has both positive and negative loadings suggesting that urinary carnosine levels (U-CAR) positively correlates with urinary concentration of carnosine adducts (U-PAL and U-POL). This strong correlation was emphasized after VARIMAX factors rotation as reported in Table 1. Interestingly, the individual PC2 scores showed no significant values (score >2) for any subject in the control group, while after the intervention some significant scores were found for three treated subjects (i.e. #9, #13 and #26).

**Figure 4:**
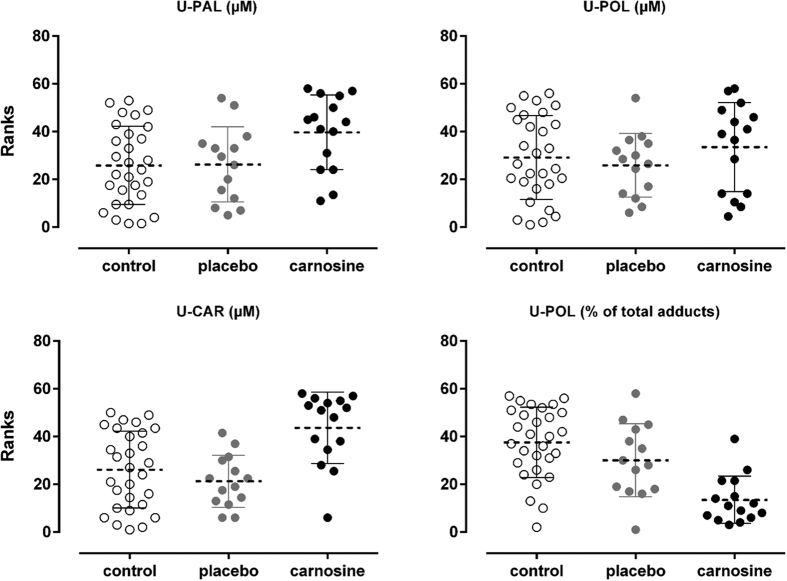
Kruskal-Wallis test Ranks resulting from the comparison of urinary concentrations of carnosine (U-CAR), carnosine-propanol (U-POL) and carnosine propanal (U-PAL) as well as the amount of adducts in reduced form (% of U-POL). White dots for control group individuals (n = 29), grey dots for placebo group individuals (n = 14) and black dots for carnosine group individuals (n = 15). Dashed lines represent the mean rank values for each experimental group.

In order to better understand the PCA results, statistic tests were also performed independently for each single variable looking for the differences among the groups. We observed a huge increase of sample variance since the mean concentration of carnosine was 71.06 ± 110.5 μM for the carnosine group, whereas a mean of 9.03 ± 6.4 μM and 6.61 ± 3.8 μM were found for the control and placebo groups, respectively. Shapiro-Wilk Test indicated a data distribution no longer Gaussian for treated subject after carnosine administration. In detail a skewness value of 2.03 was found for the carnosine group, whereas a value of 0.38 and 0.59 was found for the control and placebo groups, respectively. This effect was due to the contribution of a few subjects showing a massive increase of urinary carnosine concentration after the intervention. For this reason analysis of variance was performed using Kurskall-Wallis nonparametric test with Dunn’s multiple comparison test for group comparison. Results indicate that treated subjects had a significant increase of U-CAR compared to both control and placebo groups as reported in Fig. 4. Adjusted p values were p = 0.0033 (treated vs control) and p = 0.0011 (treated vs placebo).

**Figure 5:**
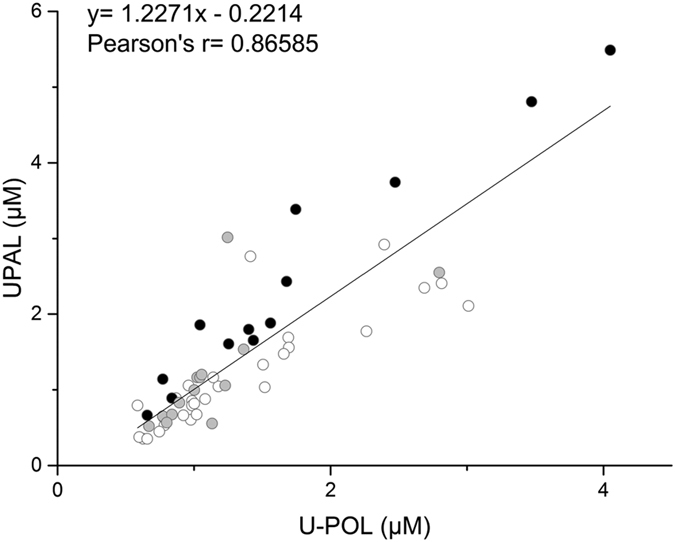
Correlation between urinary concentration of carnosine-propanol (U-POL) and carnosine-propanal (U-PAL) in the urine of 29 subjects. White dots for control group individuals, grey dots for placebo group individuals and black dots for carnosine group individuals.

As reported in Fig. 4, the carnosine group exhibited also a significant increase of carnosine-propanal adduct compared to the control group (p = 0.0291) but the significance compared to the placebo group was only borderline (p = 0.0948). Nevertheless, the concentration of the corresponding reduced adduct (i.e. carnosine-propanol) was not significantly different in treated subjects, although it showed an increasing trend. Interestingly, we observed a strong correlation between adducts concentrations as reported in Fig. 5. Moreover, the slope of the regression line (1.2271) suggests an almost one to one U-PAL/U-POL ratio with an overall slightly prevalence of adducts in non-reduced form (U-PAL). Moreover, Fig. 5 shows that treated subjects all lie above the regression line, while the placebo and control groups are more or less evenly distributed, suggesting that the carnosine group might have a lower concentration of adducts in reduced form (U-POL). As reported in Fig. 4, we confirmed that treated subject showed a significant lower percentage of adducts in the reduced form (U-POL/total adducts) compared to both the control and placebo groups (P < 0.0001 and p = 0.0244, respectively).

**Figure 6:**
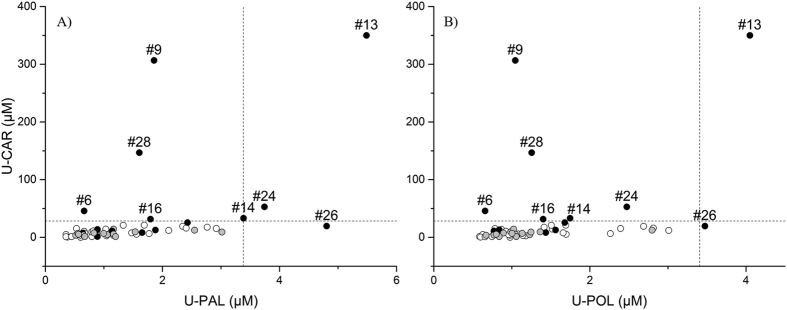
XY scatter plots reporting the urinary concentration of carnosine (U-CAR) versus the urinary concentration of carnosine-propanal (U-PAL, scatter plot **A**) or versus the urinary concentration of carnosine-propanol (U-POL, scatter plot **B**). White dots for control group individuals, grey dots for placebo group individuals and black dots for carnosine group individuals. Dashed lines define the limits including 99.7% of the observations expected for the control group (μ ± 3σ).

Finally, we were able to characterize a set of different response patterns in treated subjects by a cutoff criterion. Firstly, we calculated the concentration range including the 99.7% of the observations expected for the control group (n = 29), then we used such ranges to highlight subjects having a significant increase of either carnosine or adducts as reported in Fig. 6. The ranges correspond to a variation of 3 standard deviations from the control group mean (μ ± 3σ) and were 0–28 μM for carnosine, 0–3.42 μM for carnosine propanal (U-PAL) and 0–3.42 μM for carnosine propanol (U-POL).

In detail, none of the subjects had concentrations out of the cutoff range either in the control or in the placebo group. Conversely 46.6% of subjects receiving carnosine (7 out of 15) showed a significant increase of U-CAR levels (i.e. subject #6, #9, #13, #14, #16, #24 and #28). Among these, subject #24 also had an increased carnosine propanal level (U-PAL), while subject #13 was characterized by an increased concentration for both adducts. Subject #26 was the only one with normal U-CAR levels but increased urinary concentration of both acrolein adducts.

These response patterns could be due to individual differences in metabolism response and dietary carnosine content. Moreover, it should be pointed out that an increase of carnosine adducts is expected only in case of an overproduction of reactive carbonyl species. However, obesity is not necessarily characterized by an increased production of RCS, although it is a risk factor since the more fatty acids there are the greater the possibilities to produce lipid peroxidation products. In this context, we didn’t screened the basal level of RCS for each subject enrolled and so different response patterns are expectable.

### Parameters determined in plasma samples of overweight/obese individuals

The samples were grouped as reported above. The mass spectrometric method described in the method section “ESI-MS analysis to profile human plasma albumin” allowed the calculation of the percentage of circulating albumin in the reduced form (HSA-SH), as cysteine conjugate (HSA-SCys) and as glucose conjugate (HSA-NGlc). Total plasma protein (P-PRO), advanced glycoxidation end-products (P-AGE), advanced oxidation protein products (P-AOPP), protein carbonyls (P-PCO) and carboxymethyl-lysine (P-CML) were measured by spectrophotometric methods (see method section). The results are reported in Table S4 (supplementary information).

The correlations between variables was firstly studied using multivariate statistics to obtain information about the correlations among the concentration of the analytes. Principal Component Analysis output gave a four-component solution explaining 78% of total data variance, with Principal Component 1 (PC1) alone explaining 29% of the total variance. PC1 shows a strong negative correlation between reduced albumin and its conjugated forms (Table 2). This result is consistent with data already reported by several authors^19^,^20^,^21^,^22^. PC2 explains 21% of the total data variance and suggests that circulating levels of CML (P-CML) positively correlate with the total protein content (P-PRO) and protein carbonyls (P-PCO). PC3 explains 15% of the total data variance and describes the samples variability due to different content of P-AOPP. Finally, PC4 describes the samples variability due to different content of P-AGE. Nevertheless, the individual scores don’t suggest any particular response pattern associated with carnosine intake for the screened subjects. The same result was obtained performing independent statistical tests for each single variable, looking for the differences among groups. Analysis of variance with Kurskall-Wallis nonparametric test and Dunn’s multiple comparison test found no significant differences among groups for any of the parameters measured in the serum.

## Discussion

To our knowledge this is the first intervention study based on CAR supplementation in overweight/obese individuals. The bioavailability of carnosine and its effect on oxidative stress and AGE/ALE formation was evaluated in both urine and serum samples and in particular the following biomarkers was measured: AGE (urine and plasma), CML (plasma), PCO (plasma), AOPP (plasma), HSA isoforms (plasma), CAR (urine), AGE (urine and plasma) and CAR-acrolein adducts (urine).

In serum, no significant differences were observed among the experimental groups for any of the measured parameters. In particular carnosine and related RCS adducts were below the LLOD in all samples. This finding is consistent with previously reported data showing that carnosine is unstable in serum because of molecule hydrolysis operated by a specific enzyme (i.e. circulating carnosinase 1)^13^,^23^. Moreover, it has been demonstrated that prolonged carnosinemia can be induced by carnosinase saturation in subjects with low carnosinase activity after a 4.2 ± 0.6 g carnosine acute intake, which is higher than the dose we used^24^.

On the other hand, carnosine and two carnosine-acrolein adducts were detected in urine of all subjects both before and after carnosine supplementation. The adduct identification in urine is consistent with data already published by Baba and colleagues, reporting that carnosine-acrolein adducts were found as the most abundant carnosine byproducts in the urine of healthy non-smokers^17^. In our study it is noteworthy that carnosine adducts were found both before and after carnosine supplementation, and a significant reduction of the mean values was observed only for a set of six young volunteers after three days of strict ovo-lacto-vegetarian diet. Notably, adducts concentration were positively correlated to carnosinuria also in non-supplemented subjects, suggesting that also dietary carnosine contributes to acrolein detoxification. In the overall overweight population, we noticed a high subject-to-subject carnosinuria variability that even increased after carnosine intake. Surprisingly, only about half of the carnosine-supplemented subjects showed significantly increased carnosinuria. Among these, only some subjects showed higher levels of carnosine adducts and only a single individual showed increased excretion of adducts but normal carnosinuria. The individual variability in the response to carnosine supplementation could be influenced by several factors including carnosine dietary intake, metabolism and the basal production of reactive carbonyl species. However, further experiments are required to investigate the factors contributing to the observed variability.

Interestingly, our findings also suggest that carnosine supplementation is able to significantly reduce the percentage of excreted adducts in reduced form (54.70% ± 6.9 for control, 51.18 ± 8.4% for placebo and 43.78 ± 5.5% for carnosine). However, further investigations are needed to clarify the mechanism leading to such a change, although a possible explanation is that the increase of adducts concentration challenges the metabolic systems responsible for adduct reduction^17^.

Although carnosine experimentation in humans have been limited so far by its low bioavailability, the findings herein reported demonstrate that carnosine is able to trap acrolein *in vivo* despite undetectable plasma concentration. Moreover, the adducts herein described are consistent with the RCS trapping mechanism previously described *in vitro* and in animal models that demonstrated the efficacy of carnosine and some analogues with improved activity and better metabolic stability^13^,^16^,^25^,^26^,^27^.

Nevertheless, more investigations are needed to clarify the biological mechanisms underlying our findings, especially concerning the positive effects on glucose tolerance and insulin sensitivity observed after three months-carnosine-treatment in individuals with prediabetes within the same population described in this study^28^. However, the results herein reported can be considered an important step for the development of promising therapeutic agents able to reduce the RCS-mediated damage in humans, since they demonstrate that carnosine and histidine dipeptides in general are able to trap acrolein in obesity.

## Materials and Methods

### Chemicals and Reagents

HPLC-grade water was prepared with a Milli-Q water purification system of Millipore (Milan, Italy).

Acrolein, perfluoropentanoic acid, formic acid and all the other reagents and organic solvents mentioned (chromasolv^®^ or analytical grade) were from Sigma-Aldrich (Milan, Italy). Carnosine (beta-alanyl-L-histidine) and tyrosyl-histidine (TH) were a generous gift from Flamma S.p.A (Chignolo d’Isola, Bergamo, Italy). 4-hydroxy2-nonenal (HNE) was prepared by hydrolysis from its corresponding diethylacetal and its concentration determined by UV spectroscopy (λ_max_ = 224 nm; ε = 13750 cm^−1^ M^−1^) as previously described^29^.

### Carnosine supplementation study design

This was a single-center, randomized, double blind, placebo-controlled intervention study of carnosine supplementation to overweight/obese individuals. The daily dose of carnosine was 2 g divided into two 1 g doses, administered orally in the morning and in the evening for 12 consecutive weeks. The study protocol was approved by the local Ethics committee of University Hospital Bratislava, and it conforms to the ethical guidelines of the Helsinki declaration of 2000. All individuals signed a written informed consent prior study entry. There was one drop-out from the study due to non-compliance. No side effects were reported during the course of the study^30^.

The study population consisted of 29 overweight to obese non-vegetarian sedentary individuals, 8 females and 21 males, age 42.62 ± 7.37 years; BMI 31.80 ± 4.12 kg/m^2^. Volunteers did not receive any regular medication nor food supplements and were asked to refrain from substantial changes in their lifestyle habits in the course of the study. Prior to blood sampling, participants were asked to abstain from strenuous exercise, alcohol and caffeine for 3 days.

### Biological specimen collection

For the subjects involved in the carnosine supplementation study, the samples of blood and urine were taken in the morning at 8 o’clock, after a 12-h overnight fast, before and after 12-week carnosine supplementation. The samples of urine were centrifuged for 10 min at 4 °C, 400 × g. Plasma samples were taken into pre-cooled sample tubes containing EDTA and immediately spun down for 10 minutes, 1600 × g, at 4 °C to obtain plasma as supernatant.

Blood and urine samples were collected also from six healthy volunteers (four males, two females, 25.67 ± 2.06 years old, BMI 21.63 ± 2.34) after a three-day ovo-lacto-vegetarian diet, pooled and used as blank matrix for the calibration curves. Blood was collected by venipuncture with Terumo venosafe K_2_EDTA tubes (CEA, Milan, Italy) and centrifuged for 10 min at 1000 × g at 20 °C to obtain plasma as supernatant. Urine samples were pooled and centrifuged for 10 min at 4 °C, 400 × g. All samples were stored at −80 °C until their use.

### Preparation and purification of carnosine adducts

The reactive aldehydes used and the structure of their corresponding adducts with carnosine are reported in Fig. 1.

For each aldehyde we prepared a reaction batch in 100 mM sodium phosphate dibasic buffer pH 7.4 containing 10 mM carnosine. The reaction was started by spiking the batch with 1 mM reactive aldehyde. After an overnight incubation at 37 °C the batch was split into two aliquots and one was stored immediately at −20 °C to stop the reaction. The second aliquot was treated with 100 mM NaBH_4_ for 30 minutes at room temperature in the dark to stop the reaction by reducing both the unreacted aldehyde and adducts^9^, this aliquot was then stored at −20 °C until use.

The total amount of reactive aldehyde consumed in each batch was calculated by a LC-UV method, as reported by Vistoli and colleagues^31^.

The characterization of adducts and their relative abundance was performed by LC-ESI-MS using the method described in the section “Qualitative method for carnosine adducts identification by LC-ESI-MSMS”.

For a reaction producing n adducts, the concentration of each adduct was calculated by the mass balance reported in equation (1) and considering the relative abundance of adducts proportional to the relative abundance of the chromatographic peak areas detected by LC-ESI-MS.


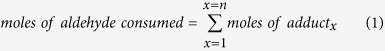


The concentration of adducts stock solution stored at −20 °C was checked each time before the use by the LC-ESI-MS method, to ensure that the concentrations of the original solutions were within the limits of the maximum established error (≤5%).

### Carnosine and adducts calibration curves in blank plasma and urines

The blank specimens were checked by the applied analytical procedure (see method section “Carnosine and adducts quantitation by LC-ESI-MS”) to ensure they did not contain any adduct above the FDA prescribed limit, corresponding to an amount below 20% of the LLOQ^32^. Calibration samples were prepared by spiking carnosine in blank matrices at the following final concentrations: 0.5, 1, 5, 25, 50, 100, 200 μM for urine and 0.1, 0.5, 1, 5, 10, 20, 50, 100 μM for plasma. The internal standard (i.e. TH) was added at a final concentration of 35 and 5 μM for urine and plasma, respectively.

Calibration samples for adducts were prepared by spiking blank urine samples with each adduct working solutions to provide the following final concentrations: 0.1, 0.8, 1.6, 4.8, 9.6 μM for carnosine propanal and 0.2, 1.6, 3.2, 9.6, 19.2 μM for carnosine-propanol. The internal Standard (i.e. TH) was added at a final concentration of 1 μM.

Before LC-MS analysis the urine samples were diluted 1:1 with 4% (v/v) aqueous TCA and then spiked with the internal standard. In order to remove the particulate matter, each sample was then spun at 14.000 g for 10 minutes by using a Thermo Heraeus Megafuge refrigerated centrifuge (Thermo, Milan, Italy). The supernatant was then collected and aliquoted in a plate well, ready to be injected by the chromatograph.

Plasma samples were firstly deproteinized by adding 5-sulfosalicilic acid at a final concentration of 0.18 M (about 4% w/v) and keeping the samples for 5 minutes at 5 °C. The samples were then spun as described above and the supernatants diluted 1:10 with 5 mM aqueous PFPA and spiked with the TH.

Three independent samples were prepared for each concentration tested and the analyses were done in triplicate. The calibration curves were built by the least square linear regression obtained by plotting the nominal concentration of the analytes (x axis) versus the analytes/internal standard peak area ratio (y axis).

### Sample preparation

All the specimens collected from overweight subjects were processed as described for the calibration curve samples (see method section “Carnosine and adducts calibration curves in blank plasma and urines”). The only variation was for urine samples that were first centrifuged without further dilution, and then spiked with the internal standard.

### Analytical methods

#### Qualitative method for carnosine adducts identification by LC-ESI-MSMS

The identification and characterization of carnosine adducts was performed by means of a high resolution mass spectrometer (LTQ Orbitrap XL, Thermo, Milano, Italy) operating in data-dependent scan mode.

A Dionex UltiMate 3000 RSLCnano System (Thermo, Milan, Italy) was employed for solvent and sample delivery. Separations were performed by a Phenomenex Polar analytical column (4 μm particle size, 2 mm i.d., 150 mm length) protected by a Phenomenex Polar RP guard column (4 μm particle size, 2 mm i.d., 4 mm length). The elution of the analytes was achieved by a multi-step binary gradient at a flow rate of 0.2 mL/min, using diluted PFPA (0.08% in water) as mobile phase A, and pure acetonitrile as mobile phase B. After the injection five minutes of isocratic flow at 99% buffer A and 1% buffer B was used for sample loading and matrix interferents removal, keeping the flow diverted from the electrospray source for the first 3 minutes. Buffer B percentage was then increased up to 80% in 24 minutes and kept constant for 5 minutes. The composition of the eluent was then restored to 100% A within 1 min, and the system was re-equilibrated for 5 min. Sample injection was done in full loop mode (20 μL) and the samples rack was maintained at 4 °C.

The LC system was connected to the mass spectrometer through a Finnigan IonMax ESI source operating in positive ion mode as follows: capillary temperature 275 °C; spray voltage 4.5 kV, tube lens 100 V, skimmer lens 5 V, capillary 40 V. Nitrogen was used as nebulizing gas at the following pressure: sheath gas 35%; auxiliary gas 10%.

During analysis, a LTQ-Orbitrap XL mass spectrometer continuously performed scan cycles including five events. The first event was a full scan acquisition in a 150–1000 m/z range, using the Orbitrap analyzer in profile mode, and a resolution of 30000 (FWHM at m/z 400). Lock mass option was enabled to provide real time internal mass calibration during the analysis using as reference a list of 20 abundant and known background signals already reported by Keller and colleagues as common air contaminants in mass spectrometry^33^.

The events from 2 to 4 were done by the ion trap, which recorded the MSMS spectra for the 3 most intense ions exceeding the intensity of 5 × 10^4^. MSMS spectra were generated both by CID and HCD fragmentation modes (isolation width, 3 m/z; normalized collision energy, 50 CID arbitrary units, 45 HCD arbitrary units). Dynamic exclusion was enabled to avoid the acquisition of redundant MSMS data (repeat count, 2; repeat duration, 30 s; exclusion list size, 500; exclusion duration, 60 s; relative exclusion mass width, 10 ppm). Charge state screening and monoisotopic precursor selection was enabled, quadruply and unassigned charged ions were not fragmented.

The fifth event was set as a targeted MSMS scan using CID with the settings reported above. The m/z values for precursor ion to select for MSMS fragmentation are reported in Table S5.

Instrument control was provided by the softwares Xcalibur and Chromeleon Xpress (version 2.07 and 6.80, respectively, Thermo Fisher Scientific, Rodano, MI, Italy).

#### Carnosine and adducts quantitation by LC-ESI-MS

The urinary and plasma levels of carnosine and corresponding adducts were measured by means of a triple quadrupole mass spectrometer (TSQ quantum ultra, Thermo, Milano, Italy) operating in multiple reaction monitoring (MRM).

A Surveyor LC system (Thermo, Milan, Italy) was employed for solvent and sample delivery. Separations were performed by a Phenomenex Polar analytical column (4 μm particle size, 2 mm i.d., 150 mm length) protected by a Phenomenex Polar RP guard column (4 μm particle size, 2 mm i.d., 4 mm length). The elution of the analytes was achieved by a multi-step binary gradient at a flow rate of 0.2 mL/min, using diluted perfluoropentanoic acid (0.08% in water) as mobile phase A, and pure acetonitrile as mobile phase B. After the injection five minutes of isocratic flow at 99% buffer A and 1% buffer B was used for sample loading and matrix interferents removal, keeping the flow diverted from the electrospray source for the first 3 minutes. Buffer B percentage was then increased to 30% in one minute and kept constant for 3 minutes, followed by another buffer B increase up to 60% in one minute. Buffer B was kept at 60% for one additional minute before restoring the mobile phase composition to the initial condition to equilibrate the column before the next injection. The equilibration time was 5 minutes. Sample injection was done in partial sample loop mode. The injection volumes were 10 μL for urine samples (20 μL sample loop) and 50 μL for plasma supernatants (100 μL sample loop). The samples rack was maintained at 4 °C.

The LC system was connected to the mass spectrometer through a Finnigan IonMax ESI source operating in positive ion mode. ESI interface parameters were set as follows: capillary temperature 275 °C; spray voltage 4.5 kV, tube lens 100 V, skimmer lens 5 V, capillary 40 V. Nitrogen was used as nebulizing gas at the following pressure: sheath gas 35%; auxiliary gas 10%.

Such conditions were chosen after a tuning procedure aimed at maximizing the signal intensities of the protonated molecules [M + H]^+^ and MRM transitions summarized in Table S6 were optimized in order to allow the maximum sensitivity.

The other parameters optimized were: argon gas pressure in the collision Q2 (1.5 mbar); scan width for Q1 and Q3 (0.50 m/z, same for all MRM channels); scan rate (dwell time, 0.1 s/scan); fragmentation was done using CID mode (isolation width, 0.5 m/z; normalized collision energy, 28 CID arbitrary units).

Full instrument control and extraction of peak areas used for quantitation were provided by Xcalibur software (version 2.0.7, Thermo Fisher Scientific, Rodano, MI, Italy).

#### ESI-MS analysis to profile human plasma albumin

The analyses were performed by an automated loop injection method, developed on the same analytic platform used for carnosine adduct quantitation (see above).

Before the analysis, plasma was centrifuged at 14.000 g for 10 min at 4 °C for removal of particulate matter and then diluted 200 fold into H2O/CH3CN/HCOOH (70/30/0.1; v/v/v) similarly to the conditions reported by Beck and colleagues^34^. Samples were then centrifuged again for 10 minutes at 18000 × g and the supernatant was placed in clear glass vials and kept at 4 °C in the autosampler compartment. Sampling was programmed as a 10 μL partial-loop injection performed by the LC system. Once loaded into the sample loop, samples were pushed at a flow rate of 10 μL/min through peek tubing directly connected to the ESI source. The mobile phase was delivered by the pump as an isocratic binary gradient at the final composition H_2_O/CH_3_CN/HCOOH (70/30/0.1; v/v/v). The analyzer was operating in conditions similar to the ones reported by Regazzoni and colleagues^19^.

Briefly, MS spectra were acquired for 10 minutes by a TSQ Quantum Ultra mass spectrometer in positive ion mode using the following settings: scan range m/z 1400–1500, 3 micro scan per scan, peak full width at half-maximum (FWHM), 0.50 m/z at Q1 and Q3, capillary temperature 275 °C, spray voltage applied to the needle 4.0 kV; capillary voltage 40 V; tube lens voltage 200 V; nebulizer gas (nitrogen) flow rate set at 10%. The percentage of each albumin isoform was calculated as reported by Regazzoni and colleagues^19^.

Full instrument control and extraction of albumin ESI mass spectra were provided by Xcalibur software (version 2.0.7, Thermo Fisher Scientific, Rodano, MI, Italy). Mass spectra deconvolution was provided by MagTran software version 1.02^35^.

#### Fluorescence and UV-vis assays for urine and plasma quantitation of total protein, Advanced Glycoxidation End-Products (AGE), Advanced Oxidation Protein Products (AOPP), Protein Carbonyls (PCO), Carboxymethyl-lysine (CML) and creatinine (CRE)

Fluorescence and UV-vis assays were used for determine the content of Advanced Glycation End products (AGE), creatinine and protein in urine.

Creatinine concentration in urine was measured by a colorimetric assay based on Jaffe reaction (Cayman, USA). The urine samples are diluted 1:10 with H_2_O and then treated with an alkaline picrate solution. When the creatinine reacts with the picrate a yellow/orange coloration is formed. The color intensity is proportional to creatinine concentration and it is measured at 500 nm. The sample creatinine concentration is determined using a creatinine standard curve. The color derived by creatinine is then destroyed at acidic pH and the intensity is measured again as a factor of correction. The protocol followed is provided by the manufacturers and is based on a paper by Heinegard and colleagues^36^.

Total protein concentration in urine was calculated by means of Bradford assay, following the procedure reported by Bradford and by Zor and colleagues^37^,^38^. In plasma the same assay was used following the procedure reported by Carini and colleagues and by Aldini and colleagues^10^,^11^.

Advanced Glycoxidation End-Products (AGE) were measured by a fluorimetric assay. The samples were diluted with a physiological solution (0.9% NaCl) and the fluorescence was measured by a Perkin Elmer LS50B fluorimeter (Perkin Elmer, Lissone, Italy). Urine and plasma samples were diluted 20 folds and 50 folds, respectively. The excitation λ was set at 370 nm with a slit of 5 nm and the emission λ was set at 440 nm with a slit of 5 nm.

Plasma AOPP were quantified following the protocol reported by Witko-Sarsat and colleagues^39^. Briefly an aliquot of plasma was diluted five folds with PBS solution, then 200 μL of the diluted plasma was placed in a well. The sample was spiked with 20 μL of concentrated acetic acid, then with 10 μL of a 1.16 M potassium iodide solution, and finally with 20 μL of concentrated acetic acid. The treated solution was read at a λ of 340 nm by a Wallac Victor2 multilabel counter, Perkin Elmer (Perkin Elmer, Lissone, Italy).

Serum protein carbonyls were measured by a PCO assay based on 2,4-Dinitrophenylhydrazine (DNPH) reaction. The protocol followed is described by Levine and colleagues^40^.

Plasma CML levels were quantified by OxiSelect™ Nε-(carboxymethyl) lysine (CML) Competitive ELISA Kit (Cellbiolabs, Milan, Italy) and the procedure followed is described by Reddy and colleagues^41^.

### Data analysis and statistics

Mass spectra elaboration was provided by Xcalibur software (version 2.0.7, Thermo Fisher Scientific, Rodano, MI, Italy). Mass spectra deconvolution was provided by MagTran software version 1.02^35^. Data analysis and statistics were provided by Prism (version 6.0 Graphpad Software Inc, USA), Origin (version 9.1 Originlab Corp, USA) and the Microsoft^®^ Excel^®^ macros Multibase (Numerical Dynamics, Japan) and RealStatistics (Release 3.5, Copyright 2013–2015 Charles Zaiontz. www.real-statistics.com).

## Additional Information

**How to cite this article**: Regazzoni, L. *et al*. A carnosine intervention study in overweight human volunteers: bioavailability and reactive carbonyl species sequestering effect. *Sci. Rep.* **6**, 27224; doi: 10.1038/srep27224 (2016).

## Supplementary Material

Supplementary Information

## Figures and Tables

**Table 1:** PCA loadings for the first two components (urinary parameters) before and after VARIMAX rotation.

Attribute	original		rotated	
	PC1	PC2	PC1	PC2
Variance explained (cumulated value)	50% (50%)	22% (72%)	37% (37%)	35% (72%)
U-PAL	**0.85**	0.41	**0.90**	0.27
U-POL	**0.85**	0.32	**0.84**	0.35
U-CRE	**0.77**	−0.26	0.39	**0.71**
U-PRO	**0.70**	**−0.50**	0.17	**0.84**
U-AGE	**0.51**	**−0.64**	−0.06	**0.81**
U-CAR	0.45	**0.57**	**0.72**	−0.11

Strong communalities in bold.

**Table 2:** PCA loadings for the first four components (plasma parameters).

Attribute	PC1	PC2	PC3	PC4
Variance explained (cumulated value)	29% (29%)	21% (50%)	15% (65%)	13% (78%)
HSASH	**−0.96**	−0.16	0.10	−0.14
HSACys	**0.86**	0.10	−0.30	0.30
HSAGlc	**0.61**	0.24	0.44	−0.37
P-PRO	−0.17	**0.79**	−0.03	−0.22
P-PCO	−0.10	**0.65**	−0.35	0.39
P-CML	−0.46	**0.61**	−0.01	0.09
P-AOPP	−0.16	−0.37	**−0.75**	−0.11
P-AGE	0.19	0.18	−0.42	**−0.76**

Strong communalities in bold.
