# Supplementary material for: A carnosine intervention study in overweight human volunteers: bioavailability and reactive carbonyl species sequestering effect
Source: Sci Rep. 2016 Jun 6;6:27224. doi: 10.1038/srep27224 (PMC4893669; doi:10.1038/srep27224)
Supplement: Supplementary Information [file srep27224-s1.pdf]

A carnosine intervention study in overweight human volunteers: bioavailability and reactive carbonyl species sequestering effect

Luca Regazzoni[a*], Barbora de Courten[b*], Davide Garzon[a], Alessandra Altomare[a], Cristina Marinello[a], Michaela Jakubova[d], Silvia Vallova[d], Patrik Krumpolec[c], Marina Carini[a], Jozef Ukropec[,c], Barbara Ukropcova[c,d^^] and Giancarlo Aldini[a^]

[a] Department of Pharmaceutical Sciences, Università degli Studi di Milano, Milan, Italy

[b] Monash Centre for Health, Research and Implementation, School of Public health and Preventive Medicine, Melbourne, Australia

[c] Institute of Experimental Endocrinology, Biomedical Research Center, Slovak Academy of Sciences, Bratislava, Slovakia

[d] Faculty of Medicine, Comenius University, Bratislava, Slovakia

Supplementary material

Adduct type	**HNE**	**ACR**
	Adduct concentration (µM)	
Michael adduct	497	230
Schiff base (aldimine)	453	295
FDP-CAR	-	415
MP-CAR	-	10

Table S1- Concentration of the carnosine adducts.

analytical method for urine

Concentration (µM)	Intraday (CV %)	Interday (CV%)	Accuracy
1	8.6	10.1	± 9.3%
25	5.3	11.3	± 5.1 %
200	6.1	6.8	± 4.7%

analytical method for plasma

Concentration (µM)	Intraday (CV %)	Interday (CV%)	Accuracy
0.5	9.7	12.3	± 8.8%
10	6.6	10.5	± 6.3 %
100	4.9	5.8	± 4.1%

Table S2- Intra and inter-day precision and accuracy data for carnosine in urine and plasma

Subject #	group	U-CRE	U-PRO	U-AGE	U-PAL	U-POL	U-CAR
		mg/mL		UF/mL	nmoles/mL		
# 1	control	0.03	1.21	948.02	0.60	0.98	0.09
# 2	control	0.08	8.19	2141.46	0.89	0.87	13.70
# 3	control	0.07	3.93	5210.06	0.79	0.59	1.38
# 4	control	0.08	12.99	1078.70	0.35	0.63	4.95
# 5	control	0.09	2.61	1009.23	0.85	0.99	8.84
# 6	control	0.11	4.88	2487.92	0.53	0.78	15.32
# 7	control	0.15	5.69	1312.12	0.60	0.98	3.65
# 8	control	0.13	9.18	2412.23	2.11	3.01	12.08
# 9	control	0.14	7.37	2655.77	1.69	1.69	21.15
# 12	control	0.13	8.26	1982.79	2.76	1.41	17.58
# 13	control	0.09	6.34	1874.86	2.35	2.69	19.20
# 14	control	0.20	8.38	1995.39	1.56	1.70	5.27
# 15	control	0.02	0.63	245.49	0.38	0.60	0.50
# 16	control	0.16	17.41	2856.87	1.03	1.52	14.34
# 17	control	0.06	3.85	2695.11	0.66	0.92	10.46
# 18	control	0.13	6.84	1579.98	1.06	0.96	4.62
# 19	control	0.11	13.28	1947.14	0.88	1.08	5.92
# 20	control	0.19	7.99	3260.62	1.77	2.26	6.57
# 21	control	0.19	6.93	4144.75	1.33	1.51	20.82
# 22	control	0.07	6.90	1178.90	1.04	1.18	2.68
# 23	control	0.10	1.60	965.19	1.16	1.14	3.33
# 24	control	0.07	2.11	1161.95	0.79	0.99	9.48
# 25	control	0.10	18.57	2147.52	0.67	1.02	7.22
# 26	control	0.25	13.18	2218.11	2.41	2.81	15.96
# 27	control	0.03	1.90	452.89	0.35	0.66	1.06
# 28	control	0.09	14.29	1463.01	1.47	1.66	7.86
# 29	control	0.06	4.74	534.19	0.45	0.75	1.38
# 30	control	0.18	7.56	1750.71	0.82	1.00	11.10
# 31	control	0.17	11.51	2560.08	2.92	2.39	15.32
# 1	carnosine	0.05	3.12	928.93	0.89	0.84	1.38
# 2	placebo	0.13	7.68	1637.53	1.16	1.02	14.34
# 3	placebo	0.10	5.45	3766.44	0.52	0.67	3.65
# 4	carnosine	0.05	3.37	1957.97	1.14	0.77	11.10
# 5	carnosine	0.05	2.46	641.13	0.64	0.78	7.54
# 6	carnosine	0.06	4.07	1216.31	0.66	0.66	45.77
# 7	carnosine	0.14	9.24	2030.79	0.89	0.84	13.05
# 8	placebo	0.18	7.97	3529.45	2.55	2.80	12.40
# 9	carnosine	0.17	7.97	2518.33	1.86	1.04	306.58
# 12	carnosine	0.15	11.33	2288.04	2.43	1.68	25.68
# 13	carnosine	0.10	11.17	2726.18	5.49	4.05	350.00

# 14	carnosine	0.22	9.50	2159.23	3.39	1.75	33.13
# 15	placebo	0.05	7.16	1290.25	0.65	0.77	3.98
# 16	carnosine	0.16	17.14	4285.56	1.80	1.40	31.51
# 17	placebo	0.08	4.96	5368.77	0.83	0.89	7.54
# 18	placebo	0.11	13.72	2536.25	1.16	1.04	5.60
# 19	placebo	0.09	13.51	1738.42	1.06	1.23	4.62
# 20	placebo	0.07	0.71	749.10	1.20	1.06	1.38
# 21	placebo	0.10	2.58	1718.45	1.00	1.00	6.89
# 22	placebo	0.04	4.56	745.40	0.67	0.84	1.38
# 23	carnosine	0.11	2.62	1471.80	1.65	1.44	8.19
# 24	carnosine	0.15	13.78	6339.33	3.74	2.47	52.90
# 25	placebo	0.18	24.53	13604.31	0.56	1.13	6.89
# 26	carnosine	0.23	19.52	3079.14	4.81	3.47	19.53
# 27	placebo	0.09	10.13	2284.25	3.01	1.25	9.16
# 28	carnosine	0.05	8.87	3370.30	1.61	1.25	146.86
# 29	carnosine	0.15	14.93	2602.29	1.88	1.56	12.72
# 30	placebo	0.07	8.92	1557.86	0.57	0.80	5.27
# 31	placebo	0.12	4.87	1495.10	1.53	1.36	9.48

Table S3 – Urinary mean concentration (n=2) for creatinine (U-CRE), total protein (U-PRO), carboxymethyl lysine (P-CML), advanced glycoxidation end-products (U-AGE), carnosine-propanal (U-PAL), carnosine-propanol (U-POL) and carnosine (U-CAR).

Subject #	**group**	**HSA -SH**	**HSA -Cys**	**HSA -Glc**	**P-PRO**	**P-CML**	**P-AGE**	**P-AOPP**	**P-PCO**
		% HSA			mg/mL	ng/mL	UF/mL	nmoles/mL	
# 1	control	58.89	22.60	18.51	64.48	3228.51	664.14	344.97	98.01
# 2	control	59.93	22.65	17.43	66.70	3266.97	657.66	125.40	107.39
# 3	control	55.39	26.97	17.64	69.13	3578.86	799.83	193.56	154.16
# 4	control	56.81	24.34	18.85	61.36	2571.60	518.49	317.23	154.63
# 5	control	60.95	21.14	17.92	63.47	3174.77	572.50	171.37	147.89
# 6	control	58.37	22.49	19.15	69.17	3141.01	649.51	174.31	152.87
# 7	control	53.93	28.20	17.87	59.90	3348.41	618.17	296.51	155.74
# 8	control	59.39	23.12	17.50	70.18	3381.27	610.57	239.31	147.38
# 9	control	62.43	21.30	16.27	58.49	6774.31	483.13	288.94	129.85
# 12	control	60.43	22.47	17.10	71.13	3785.54	939.63	389.08	165.02
# 13	control	62.24	20.95	16.83	62.62	4973.91	541.66	211.03	56.98
# 14	control	58.92	23.46	17.62	63.60	3193.36	1071.02	323.09	79.50
# 15	control	60.99	22.30	16.72	68.02	2955.47	570.01	245.55	57.82
# 16	control	59.23	23.41	17.36	46.98	2117.39	483.42	192.15	71.88
# 17	control	59.13	22.52	18.36	61.06	2604.21	720.51	263.17	82.43
# 18	control	59.40	22.04	18.55	64.72	2749.31	639.43	403.85	58.25
# 19	control	56.21	24.94	18.86	63.48	3308.58	724.94	390.40	67.29
# 20	control	58.07	23.25	18.69	62.47	2856.75	761.51	384.19	62.47
# 21	control	56.58	25.98	17.44	73.23	3220.66	653.94	309.03	71.03
# 22	control	61.04	22.23	16.74	22.67	2785.46	601.89	564.94	83.20
# 23	control	58.40	23.94	17.67	63.91	2732.79	470.38	369.40	84.36
# 24	control	59.12	24.09	16.81	58.22	2410.89	669.53	588.02	105.96
# 25	control	57.26	25.43	17.32	62.80	3175.80	454.04	472.26	105.50
# 26	control	62.02	21.34	16.65	66.90	3099.48	554.60	483.69	127.11
# 27	control	60.60	22.39	17.02	69.99	3175.45	624.31	617.31	125.98
# 28	control	60.03	22.38	17.60	68.18	2785.83	657.94	265.90	137.72
# 29	control	57.41	25.13	17.46	63.57	2949.65	940.84	346.46	129.68
# 30	control	56.92	26.80	16.28	54.47	2089.47	479.88	548.51	129.64
# 31	control	60.37	22.80	16.84	71.97	3982.10	604.55	274.93	167.69
# 1	carnosine	60.57	21.86	17.58	61.36	2994.37	623.42	229.49	110.45
# 2	placebo	60.14	23.19	16.68	61.06	3797.93	478.71	279.04	121.51
# 3	placebo	58.24	24.71	17.05	56.34	2993.91	642.84	219.16	104.23
# 4	carnosine	59.33	22.93	17.75	54.43	2329.60	428.36	251.47	93.08
# 5	carnosine	61.39	21.95	16.67	59.41	2713.25	437.85	144.96	100.40
# 6	carnosine	58.24	24.12	17.65	61.29	2588.89	605.55	237.19	101.74
# 7	carnosine	55.02	25.56	19.43	64.61	3042.48	502.02	215.80	94.33

# 8	placebo	59.96	22.77	17.28	61.25	2929.59	1010.63	255.41	99.84
# 9	carnosine	59.52	24.08	16.40	54.19	2713.84	518.06	189.67	93.21
# 12	carnosine	59.99	23.42	16.59	63.20	2673.36	592.82	482.22	96.70
# 13	carnosine	63.26	20.25	16.50	63.68	3472.47	478.24	288.47	106.35
# 14	carnosine	59.01	23.92	17.08	49.17	2066.12	1025.69	384.02	92.93
# 15	placebo	64.70	19.12	16.20	64.67	2904.98	507.01	457.86	105.41
# 16	carnosine	60.07	22.71	17.23	50.13	2128.02	483.25	220.07	98.25
# 17	placebo	59.85	22.06	18.10	57.21	2415.98	633.89	482.28	64.08
# 18	placebo	61.81	20.78	17.42	60.58	3161.06	620.95	298.05	86.02
# 19	placebo	56.23	26.52	17.26	57.07	2936.25	667.72	344.13	82.18
# 20	placebo	58.84	23.21	17.96	56.47	2601.57	769.12	369.88	76.80
# 21	placebo	57.77	25.58	16.66	57.50	2586.35	696.90	347.88	108.10
# 22	placebo	62.77	20.33	16.90	59.61	2492.29	783.28	457.80	92.99
# 23	carnosine	60.86	21.94	17.20	54.93	2483.39	670.15	468.55	112.61
# 24	carnosine	59.11	24.01	16.89	58.14	3640.73	930.82	350.58	91.86
# 25	placebo	59.11	24.02	16.87	61.66	3235.92	537.68	419.90	112.22
# 26	carnosine	62.72	20.39	16.90	57.52	2648.22	529.18	364.10	103.54
# 27	placebo	57.60	24.86	17.55	59.78	2722.98	583.45	450.74	102.82
# 28	carnosine	60.54	21.93	17.54	58.51	2907.36	653.56	297.82	91.28
# 29	carnosine	57.81	24.99	17.21	56.15	3034.35	760.83	288.05	97.70
# 30	placebo	56.35	26.59	17.06	41.98	1642.26	518.87	333.74	83.96
# 31	placebo	62.39	21.56	16.06	76.03	4087.37	732.17	609.76	137.61

Table S4 – Plasma mean concentrations (n=2) for reduced albumin (HSA-SH), cysteinylated albumin (HSA-Cys), glycated albumin (HSA-Glc), total protein (P-PRO), carboxymethyl lysine (P-CML), advanced glycoxidation end-products (P-AGE), advanced oxidation protein products(P-AOPP) and protein carbonyls (P-PCO).

Name	**abbreviation**	**Precursor ion (m/z)**
histidine	His	156.07675
histidine-acrolein (propanal) adduct	His-propanal	212.10296
histidine-acrolein (propanol) adduct	His-propanol	214.11861
3-hydroxypropyl-mercapturic acid	3-HPMA	220.06380
carnosine	CAR	227.11387
carnosine-acrolein Schiff base adduct	CAR-ACR-SB	265.12952
carnosine-acrolein Schiff base adduct (reduced form)	CAR-ACR-SB-red	267.14517
carnosine-acrolein Michael adduct (carnosine-propanal)	POL	283.14008
reduced carnosine-acrolein Michael adduct (carnosine-propanol)	PAL	285.15573
mercapturic acid-4hydroxynonenal Schiff base adduct	MA-HNE-SB	302.14205
histidine-4-hydroxynonenal adduct	His-HNE	312.19178
histidine-dihydroxynonane adduct	His-DHN	314.20743
tyrosyl-histidine	Tyr-His (IS)	319.14008
mercapturic acid-4hydroxynonenal Michael adduct	MA-HNE	320.15262
(3-formyl-3,4-dehydropiperidino) carnosine	FDP-CAR	321.15573
mercapturic acid-dihydroxynonane Michael adduct	MA-DHN	322.16827
(3-formyl-3,4-dehydropiperidino) carnosine (reduced)	FDP-CAR -red	323.17138
carnosine-4hydroxynonenal Schiff base adduct	CAR-HNE-SB-red	367.23398
carnosine-4hydroxynonenal Michael adduct	CAR-HNE-MA	383.22890

carnosine-dihydroxynonane adduct	CAR-DHN	385.24455

Table S5- List of precursor ions of the main carnosine adducts and metabolites

compound	**Precursor ion**	**Fragment ions**	**Collision energy**
	(m/z)		(%)
Internal standard (TH)	319.1	110.1, 156.1	20
Carnosine (CAR)	227.1	110.1, 122.1	30
Carnosine propanal (PAL)	283.1	110.1, 151.2	35
Carnosine propanol (POL)	285.2	110.1, 168.2	35

Table S6- MRM transitions of carnosine, carnosine adducts and internal standard used for quantitation
